# An Experimental Study on the Scalability of Recent Node Centrality Metrics in Sparse Complex Networks

**DOI:** 10.3389/fdata.2022.797584

**Published:** 2022-02-16

**Authors:** Alexander J. Freund, Philippe J. Giabbanelli

**Affiliations:** Department of Computer Science and Software Engineering, Oxford, OH, United States

**Keywords:** empirical study, node centrality, scale-free, small-world, synthetic graph generation

## Abstract

Node centrality measures are among the most commonly used analytical techniques for networks. They have long helped analysts to identify “important” nodes that hold power in a social context, where damages could have dire consequences for transportation applications, or who should be a focus for prevention in epidemiology. Given the ubiquity of network data, new measures have been proposed, occasionally motivated by emerging applications or by the ability to interpolate existing measures. Before analysts use these measures and interpret results, the fundamental question is: are these measures likely to complete within the time window allotted to the analysis? In this paper, we comprehensively examine how the time necessary to run 18 new measures (introduced from 2005 to 2020) scales as a function of the number of nodes in the network. Our focus is on giving analysts a simple and practical estimate for sparse networks. As the time consumption depends on the properties in the network, we nuance our analysis by considering whether the network is scale-free, small-world, or random. Our results identify that several metrics run in the order of *O*(*nlogn*) and could scale to large networks, whereas others can require *O*(*n*^2^) or *O*(*n*^3^) and may become prime targets in future works for approximation algorithms or distributed implementations.

## 1. Introduction

Since its emergence in the second half of the twentieth century, measuring *node centrality* has become one of the core tasks of network analysis. A plethora of measurements have been proposed, to reflect different notions of “importance” for a node in a network. For example, a node may be more central if its removal would have a noticeable impact on a network characteristic [i.e., a “vitality centrality” approach as shown by Koschützki et al. (2005)] selected by the analyst given the research context (e.g., number of components, diameter). Alternatively, a node can be deemed more essential if it is more often traversed [i.e., the “walk structure perspective” discussed in Borgatti and Everett (2020) or “Path-Based Measures” used by Saxena and Jadeja (2022)]. This leads to many specific measures based on how traversals are aggregated [e.g., summing them in closeness centrality introduced in Beauchamp (1965) or taking the maximum shortest path in eccentricity centrality as proposed by Hage and Harary (1995)], and whether all paths are counted [e.g., as in Katz centrality from Katz (1953)] or only those of the shortest length [e.g., as in betweenness centrality from Freeman (1977)]. Such node centrality measures have been applied across a wide range of domains, ranging from the identification of prominent actors in social networks [also known as “ego networks” in Everett and Borgatti (2005)] to the position of services and goods within cities (Baniukiewicz et al., 2018). Applications have shown that the initial definitions and algorithms of certain centrality measures needed to be adapted to cope with large network sizes, leading to a wealth of research in parallel algorithms (Jamour et al., 2017) and approximation algorithms (Matta et al., 2019). Node centrality measures such as betweenness have thus reached a mature status: we know when the cost of the original algorithm may not be feasible for a given analysis, and we can then switch to a parallel/approximation approach or a different measure that captures similar aspects (Singh, 2022).

In contrast to the established measures introduced in the twentieth century, there is a paucity of research regarding the scaling behavior of many of centrality methods created in since the 2000's. As shown in online repositories of centrality measures such as CentiServer (https://www.centiserver.org/centrality/list/), 23 measures were created prior to 2000 and about 380 have been proposed since then. The growing number of measures has even accelerated in the recent years, from 10 to 20 new measures annually in the early 2010's to almost 40 in 2017 and 2018, and 63 just in 2019. Recent works continue to propose such general measures (Fronzetti Colladon and Naldi, 2020). This number only accounts for the measures listed on the repository, hence it is possible that the actual list is even longer. The lack of information on the scalability of these new measures has three related consequences. First, it creates a limitation when analyzing large networks, such as biological and social networks formed of billions of nodes (Joyce et al., 2010; Ugander et al., 2011). Indeed, analysts may refrain from using a metric without knowing how long it will take, or whether the analysis would finish within a given time period. This creates a gap between the growing set of available measures and the much smaller set of options with a guaranteed computational cost. Second, there is an impact on the research landscape: if we knew that certain node centrality measures do no scale well for current applications, then we would be able to propose parallel and/or approximation algorithms to put them within reach of analysts. This is exemplified by recent works in which the realization that a new centrality measures do not scale well “beyond hundreds of thousands of vertices” is soon followed by the emergence of algorithms that are orders of magnitude faster (van der Grinten et al., 2021). More globally, efforts are underway in the network science community to use performance-oriented algorithmic techniques that allow for an efficient computation of newer node centrality measures on big network datasets (van der Grinten et al., 2020). Third, there is a growing interest in creating “integrative” measures that combine a *basket* of centrality measures (Salavaty et al., 2020; Keng et al., 2021). If analysts knew that the overall cost was driven by a few specific measures, the combination could be trimmed to remain informational while computing significantly faster.

To guide these efforts and equip analysts with measures that they can use, this paper performs an assessment of several measures introduced from 2003 to 2020. Specifically, our goal is to identify measures that may not scale well *in practice*. On the one hand, the worst case scenario may be much higher than cases encountered by analysts, thus enabling the processing of larger networks in practice than under a theoretical upper bound. On the other hand, an estimate based on random networks may turn out to be inaccurate, thus leaving analysts in the dark for the actual resource needs of the algorithm. Consequently, our empirical measures are performed on the three structural archetypes most commonly cited (Lofdahl et al., 2015; Wang et al., 2019): small-world (i.e., high clustering and low average distance), random, and scale-free networks (i.e., power-law degree distribution). By identifying scaling bottlenecks for recent measures across these three sparse networks, our empirical analysis provides a list of algorithms that are prime candidates for approximation or parallelization in future studies.

The remainder of this paper is organized as follows. In Section 2, we provide a brief summary of the 18 centrality measures empirically assessed in this paper. The equation for each measure is provided in our Supplementary Material. Since we need to generate networks with set properties to perform each measurement, the background section also covers the fundamentals of synthetic network generation. Then, Section 3 explains how we generated the networks, performed the measurements, and obtained the scaling behaviors. These scaling behaviors form the key results of this paper and are provided in Section 4, before the final discussion in Section 5.

## 2. Background

### 2.1. Recent Centrality Metrics

We selected a sample of measures with a broad applicability, developed since the mid 2000's, thus following the dichotomy of Mnasri et al. (2021) who differentiate “classic” algorithms from the “latest” ones. Consequently, we did not include measures that are highly specific to applications such as brain networks (Crofts and Higham, 2009; Joyce et al., 2010), protein-protein interaction networks (Tew et al., 2007), clinical psychology (Jones et al., 2021), or other particular domains (Zarghami and Gunawan, 2019). Indeed, analysts already have access to guidance on the use of such measures *via* several dedicated reviews (Jalili et al., 2016; Ashtiani et al., 2018; Das et al., 2018; Bringmann et al., 2019). Our objective is thus closer to the recent work of Oldham et al., which examined the behavior of 17 centrality metrics across a variety of networks (Oldham et al., 2019). While their work informs analysts on redundancy across measures (i.e., correlation of measures), our selection seeks to reveal the cost of measures before analysts embark on computing them.

The 18 measures used in this study are listed in Table 1 together with their reference and a succinct description. A complementary and deeper description is provided in our Supplementary Material, where the equation is also stated for each measure.

**Table 1:** The 18 centrality measures used in this study.

**Centrality name**	**Year**	**References**	**Based on**
Subgraph	2005	Estrada and Rodriguez-Velazquez, 2005	Eigenvalues to count close walks
Geodesic K-Path	2006	Borgatti and Everett, 2006	Number of nodes reachable *via* shortest path of bounded length
Maximum neighborhood component	2008	Lin et al., 2008	Size of the largest connected component within the direct neighbors of a given node
Density of maximum neighborhood component		Lin et al., 2008	Ratio of edges to nodes within the largest connected component between a node's neighbors
Decay		Jackson, 2010	Proximity between a given node and every other node, weighted by a decay rate
Topological coefficient	2009	Zhuge and Zhang, 2009	Average number of neighbors of a given node that are also neighbors to a different node
Lobby Index		Campiteli et al., 2013	Largest integer k such that the node has at least k neighbors with a degree of at least k.
Coreness	2010	Kitsak et al., 2010	Sum of k-shell indexes of a given node's neighbors
Leverage		Joyce et al., 2010	Degree of a node relative to its neighbors
Group		Narayanam and Narahari, 2010	Game theory, to measure the marginal increase in group influence
Wiener Index	2011	Caporossi et al., 2012	Average distance from a given node to all other nodes
K-Path		Alahakoon et al., 2011	Number of random paths of length k from all nodes that include a given node
Diffusion Degree		Kundu et al., 2011	Degree contribution of a node and its neighbors, weighted by a propagation probability
LeaderRank		Lü et al., 2011	Convergence of a random walk
Laplacian		Gutman and Zhou, 2006	Degrees of a node and its neighbors. Equivalent to using eigenvalues in the Laplacian
Local Bridging	2016	Macker, 2016	Ratio of shortest paths going through a node, modulated by its degree and degree of neighbors
VoteRank		Zhang et al., 2016	Spreading ability, measuring by the convergence of an election process between neighbors.
Heatmap	2020	Durón, 2020	Sum of distance from a node to all others (i.e., farness) and average farness of the neighbors.

*Divisions in the table emphasize publication years*.

Many of these studies (e.g., Coreness, Decay, Geodesic k-path, LeaderRank, Local Bridging, DMNC, Subgraph, Wiener Index) *do not report the time complexity or execution speed* of the algorithm. This observation should not be interpreted as a negative point for such studies, as they may have focused on the theoretical properties of the measure or the existence of a new measure “in between” existing ones, rather than emphasizing scalability. However, it means that analysts have limited indications about execution speed if they wish to apply these measures. To understand the cost structures provided by some of the algorithms, we use the following notation: *n* and *m* are the number of nodes and edges, respectively, Δ is the maximum degree, *r* is the number of nodes for which the centrality would be computed (generally used in an algorithm that seeks to find the “top *r*”), and *K* is a user-defined number of iterations or repetitions. Several of the measures have *complex cost structures*, which may be difficult to apply for analysts. This includes VoteRank which runs in $O\left(m+rlog\left(n\right)+\frac{rm^{2}}{n^{2}}\right)$, Group centrality which takes *O*(*t*(*n* + *m*)*K* + *nlog*(*n*) + *rn* + *rKm*) (*t* is a polynomial), or *k* − *path* which performs an approximation in *O*(*k*^3^ × *n*^2−2α^ × *ln*(*n*)) (where α is an approximation parameter and *k* is a function of *m* and *n*). Several measures report a simpler time complexity, such as *O*(*n* + *m*) for diffusion degree, *O*(*m* + *n* × Δ^2^) for the Laplacian, or *O*(*K* × (*n* + *m*)) for Topological. However, these costs may *assume different underlying data structures* for the network [e.g., adjacency/incidence matrix, adjacency/incidence list, compressed representations as in Besta et al. (2018)]. This is particularly the case to access a node's neighbor, which is the primary operation involved in several centrality measures (e.g., Diffusion, Leverage, LobbyIndex, MNC/DMNC). Consequently, costs may be different when using the representation offered by a common network library such as NetworkX.

The difficulty of obtaining or applying the time complexity of the algorithms is compounded by the challenge of comparing their performances empirically. Indeed, none of the eight studies employing empirical networks had a network in common (Table 2), and studies are performed using differen hardware configurations (which may not resemble today's workstations). These challenges motivate the objective of this paper to provide one estimate based on practical differences between networks (small-world, scale-free, random), based on one experimental set-up. The next sub-section details why the estimate is provided as a function of the number of nodes.

**Table 2:** Density of networks used in previous experimental assesssments of centrality measures.

**Centrality**	**Network**	**Number of nodes n**	**Number of edges m**	**Density**
Group Centrality	Western States Power Grid	4,940	6,594 undirected	0.0005
	Collaborations in astrophysics	16,705	121,251 undirected	0.0008
K-Path	Kazaa file sharing	2,424	13,354 undirected	0.0045
	SciMet citations	2,729	10,416 undirected	0.0027
	Co-authorships in condensed matter	23,133	186,936 undirected	0.0007
	Citations (Cit-HepPh)	34,546	421,578 directed	0.0007
	Company emails at Enron	36,692	367,662 undirected	0.0005
	Social (Epinions1)	75,879	508,837 directed	0.00008
	Social (Slashdot0922)	82,168	948,464 directed	0.00014
Heatmap	University emails in Spain	1,133	5,451 undirected	0.0085
	Hyperlinks in US political blogs	1,222	16,714 undirected	0.0224
	US Airline Flights in 2010	1,572	17,214 undirected	0.0139
	Facebook from UC Irvine students	1,893	13,835 undirected	0.0077
Laplacian	Terrorist network mapped by Krebs	37	170 directed	0.1276
LeaderRank	Users of delicious.com in May 2008	1,675,008	169,378 undirected	0.0000001
Subgraph	Protein–protein interaction (yeast)	2,224	6,608 undirected	0.0026
	Protein–protein interaction (bacterium)	710	1,396 undirected	0.0055
	Words in Roget's Thesaurus of English	994	3,640 undirected	0.0073
	Words in Online Dict. of Library & Info. Science	2,898	16,376 undirected	0.0039
	Collaborations in computational geometry	3,621	9,461 undirected	0.0014
	Citations of papers on graph drawing	249	635 undirected	0.0205
	Internet at the autonomous system (1997)	3,015	5,156 undirected	0.0011
	Internet at the autonomous system (1998)	3,522	6,324 undirected	0.0010
Topological	DBLP research database	664,188	79,128 directed	0.0000001
VoteRank	Friendships of Youtube users	1,134,890	2,987,624 undirected	0.000004
	Co-authorship in condensed matters (arXiv)	23,133	93,497 undirected	0.0003
	Hyperlinks in Berkeley/Stanford webpages	685,230	7,600,595 directed	0.00001
	Hyperlinks in U. Notre Dame webpages	325,729	1,497,134 directed	0.00001

*Note that density is calculated as $\frac{m}{n\times \left(n-1\right)/2}$ for undirected networks and $\frac{m}{n\times \left(n-1\right)}$ for directed networks*.

### 2.2. The Sparsity of Complex Networks

Several of the studies listed in Table 1 followed their introduction of a new centrality measure by its empirical evaluation on a variety of networks, primarily to confirm that “important” nodes are identified. The networks used are summarized in Table 2. The *density* is defined as the ratio of edges in the network (*m*) over the number of potential edges if all nodes were connected (which depend on whether the network is directed or undirected). We observe that the density is very low for *all* networks used in experimental studies of centrality measures. Networks with such low density measures are known as *sparse*. Sparse networks are thus common when applying centrality measures, which motivates the focus of our paper on sparse networks.

For a network to be sparse, it means that the number of existing edges will always be relatively low. The range of possible number of edges is even narrower when considering scale-free networks based on the preferential attachment mechanism (Del Genio et al., 2011). Intuitively, the power-law degree distribution of scale-free networks signifies that a few nodes are very “rich” in social capital/ties, while most of the network is order of magnitudes “poorer.” If the number of edges is increased across the network, then the wealth of most nodes rises and it gradually blurs the distinction with the “rich” nodes. For this reason, scale-free networks are often studied in a narrow range of densities, occasionally called the “appropriate connection density” (Yang et al., 2013). This range is exemplified by the experiments of Duron who examined heatmap centrality on scale-free networks with a density ranging from 0.002 to 0.02 (Durón, 2020). As the number of edges is small, analysts often estimate the time consumption of a centrality algorithm solely by the number of nodes. For instance, Zhang et al. (2016) reported that “the CPU running time is 28.25 min [...] to find top-30 important nodes in network with 1,589 nodes” using another algorithm, which motivated the introduction of their proposed VoteRank centrality. Consequently, the present manuscript focuses on reporting our running time as a function of the number of nodes. Note that future examinations may also include the number of edges, as emerging results and generators are examining dense networks (c.f. Section 5).

### 2.3. Generating Networks for Benchmarking

Network algorithms are eventually destined to be applied onto real-world networks, leveraging certain properties to achieve goals such as community detection or identification of important elements *via* centrality measures. Despite the ubiquity of large network data (e.g., *via* Facebook or Twitter), there are at least three reasons for which we also employ network models to generate *synthetic* networks (Ali et al., 2014). First, applying algorithms on a set of common real-world networks helps to get an estimate of performances and compare with other solutions, but synthetic network generators allow to more comprehensively characterize an algorithm's effectiveness (e.g., accuracy of the answer) and efficiency (e.g., runtime) (Kanovsky, 2010). As summarized by Rossetti in the context of community detection algorithms, “the main rationale behind the adoption of network generators as benchmarks while analyzing the performances of a [network] algorithm lies in the ability to produce datasets that allow *controlled environment testing*.” (Rossetti, 2017) This need for benchmarking often motivated the development of synthetic generators, particularly in fields where real-world instances were scarce (Giabbanelli, 2010; Sahraeian and Yoon, 2012; Pasta and Zaidi, 2018). Second, snapshots of large real-world networks are often only samples, hence they miss links that were not expressed within a specific time window or happen between two entities in one network instead of another (Lofdahl et al., 2015). Third, highly detailed network data destined for applications such as agent-based modeling (e.g., spread of word-of-mouth based on social ties and personal characteristics) may face privacy concern at the point of access (Qin et al., 2017) (e.g., retrieving and assembling contact lists across cellphone users) or include sensitive information, which can be difficult to anonymize (Lofdahl et al., 2015).

Synthetic networks should capture the relevant structural properties of the real-world networks for which the algorithm is intended to be used, such as a certain degree distribution or clustering. At the same time, the network generators need to conserve a sufficient degree of freedom to generate networks based on properties that are unknown to algorithms' developers and may impact effectiveness or efficiency. For example, the ability to generate networks of various sizes (i.e., number of nodes) can help to identify how the runtime depends on network size (i.e., time complexity), hence assessing the *scalability* of an algorithm (Rossetti, 2017). In addition, the research community often favors generators that are relatively simple, based on only a few rules (Mei and Stefa, 2009; Sahraeian and Yoon, 2012; Wang et al., 2019). Indeed, “a simple method for generating complex networks that have specific properties of a real system is of significant importance (Wang et al., 2019).”

Competing demands are thus placed onto network generators: they need to be sufficiently flexible, such that “algorithms may be tested and compared in different conditions” (Kanovsky, 2010), but they also need to specifically capture a set of desired properties. As a result, generators are unable to generate certain combinations (e.g., some network sizes cannot be obtained) or only yield a subset of the target networks with the desired property. For example, consider hierarchical network generators in which the next iteration of a network consists of making *k* copies and adding new edges (Ravasz and Barabási, 2003; Barriere et al., 2016). A generator *A* may use a network of |*A*_0_| = 50 nodes as a start, and duplicate it at every iteration. Another generator *B* may start with |*B*_0_| = 256 nodes and make five copies. A user of *A* may thus only be able to generate networks of sizes 50, 100, 150, etc., while users of *B* will have 256, 1, 280, 6, 400, etc., nodes. This is not usually a major limitation: a user interested in determining the time complexity of a network algorithm needs to perform a few measurements on networks of different sizes, rather than a measurement for every network size. In other words, if a network of a specific size cannot be generated, that may be unimportant (that size may not have been needed) or tolerable (a similar size may suffice). A problem arises when *several* generators are used. For the generators *A* and *B*, a user who needs matching network sizes can only obtain the first one at |*A*_7_| = |*B*_2_| = 6, 400, which may be much larger than needed in an application context. The problem of limited and incompatible networks across generators is particularly salient in application areas such as simulations (Amblard et al., 2015). The number of nodes and average degree may need to match the population size and average contacts, and various network generators are used due to the uncertainty in the nature of the social ties (e.g., due to unknown mobility patterns in the population) (Giabbanelli et al., 2014). If each generator yields an even slightly different virtual population in size or average degree, then simulation results would hardly be comparable.

## 3. Materials and Methods

### 3.1. Overview

In our experimental approach, the scaling behavior of 18 node centrality metrics is computed on scale-free networks, small-world networks, and an equivalent randomized network serving as a comparison case. We primarily study scaling (i.e., wall-clock time) in relation to network size (*n*). We start with small networks of size *n* = 100 and progressively increase the network size until we reach 16, 000. We noticed in early experiments that wall-clock times for small networks (*n* = 100, 200, …, 3, 200) tended to be too similar across centrality metrics, since algorithms often run very quickly for such instances. We thus sweep the range of small networks quickly (by *doubling* in size from *n* = 100 up to *n* = 3, 200) and then devote more measurements to larger networks (by increasing in size by 1, 600 i.e., *n* = 4, 800, 6, 400, 8, 000, …, 14, 400, 16, 000). This leads to a total of 14 network sizes. To account for variation in network structure, we generate enough instances of each network type at each size to achieve a 95% confidence interval in the average wall-clock time. Given the network size (x-axis) and associated wall-clock time of a centrality measure (y-axis), we then use curve fitting methods to identify the relationship (i.e., the scaling behavior of the centrality algorithm).

Since the scaling behavior of node centrality measures also depends on the number of edges, we focus on sparse graphs and ensure that network instances match in edge density across different network types. For example, at size *n* = 800, all instances of small-world, scale-free, and random networks will have a comparable number of edges within the limitations of their respective network generators. Since big network data is often processed in the cloud or on high-performance computing clusters (HPC), we measure the computation time on an HPC with dual Intel Xeon Gold 6126 processors.

### 3.2. Network Generation

#### 3.2.1. Generators, Goals, and Constraints

A *small-world* generator creates a network with low average distance and high clustering, thus capturing the notion that individuals form groups and that a few individuals belonging to different groups act as shortcuts. The Watts-Strogatz (WS) generator starts with a cycle of *n* nodes, each connected to its *k* nearest neighbor (thus providing high clustering), then rewires edges with probability *p* to create shortcuts (hence lowering distances). Given the rewiring process, the graph may no longer be fully connected. A *scale-free* network has a power-law degree distribution, hence most nodes have a regular number of social ties while a few nodes (potentially acting as “hubs”) have a much larger number of ties. The emergence of these networks is explained by growth and preferential attachment, which are the core mechanisms of many scale-free network generators in which *n* nodes are added to the graph and wired preferentially to high degree nodes. We use the method by Bollobás et al. (2003) to create directed scale-free networks, which is governed by several parameters: the number of nodes *n*, the probabilities that a new node connects to an existing one based on its in-degree (α) or out-degree (γ), and the probability β for existing nodes to become connected. The generator is constrained such that α + β + γ = 1.

A simple *random* network exhibits neither of these properties: the clustering is low (hence not small-world) and the degree distribution is not a power-law (it may be either Poisson or binomial depending on network size). Such networks may be straightforwardly generated using the Erdős-Rényi model, in which there is a probability *p* to connect pairs of (distinct) nodes chosen among *n*. Although these models simple, we select them as representative generators due to their widespread use, including as the building blocks of synthetic graphs in recent works (Goyal et al., 2020).

The basic requirements for benchmarking or simulation is that all networks must be connected and contain no self-loops (edges between a node and itself). To ensure connectivity, we use the variation by Newman and Watts on the WS model, in which edges are added instead of being rewired (Newman and Watts, 1999). To generate the connected random network, we start by connecting the *n* nodes to form a path and then add the remaining edges as in the Erdős-Rényi model. The scale-free network being obtained by adding edges to existing nodes, it is necessarily connected. Self-loops can simply be removed after a network is created.

Our *objective* is to ensure that, for a given network size, networks generated across all three generators are also similar in edge density (the number of edges present out of the total possible number of edges). The network size and edge densities are two key characteristics that can impact the effectiveness/efficiency of algorithms and they are also often core network metrics in simulation applications.

For a given size *n*, the random network generator can straightforwardly be made to create an instance with a desired network density, by tuning the probability *p*. Given this flexibility, this generator can be used last. In contrast, the scale-free generator requires a fine tuning of three parameters (α, β, γ). It is also limited in the density that it can achieve, as a very dense network would also become small-world (which is not allowed by the generator) due to falling distances and a rising clustering. The abilities and limitations of each generator thus play a role in determining the order in which they should be triggered, and how to tune the next generator accordingly.

#### 3.2.2. Procedure

The edge density of a network can be measured easily, yet it is difficult to enforce consistent density across generators. While the small-world and random network generators allow parameters to control the number of edges, the scale-free generator does not. Subsequently, we solve this challenge at each network size through three key steps. First, we sample the edge density that can be produced by the most restrictive generator (scale-free). Then, we tune the next generator (small-world) by establishing the maximum number of neighbors *k* for each node and probability *p* of additional edges, without exceeding the average from the scale-free generator. Finally, we create the random network by using a probability *p* that produces the required density.

Our overall procedure is shown in Algorithm 1. We start by generating the *i* instances of a scale-free network required by the user and we track the number of edges across these instances. If *i* is too small, the estimated number of edges may not be reliable hence additional samples are generated to achieve a 95% Confidence Interval. In order to obtain a conservative estimate of the duration needed for our computations on a shared cluster, we set the base number of instances *i* to 100. The estimated output from the scale-free generator is used to shape the input to the small-world generator (parameters *k* and *p*) and the random network, which starts with a path (to ensure connectivity) before adding the random edges.

**Algorithm 1:**
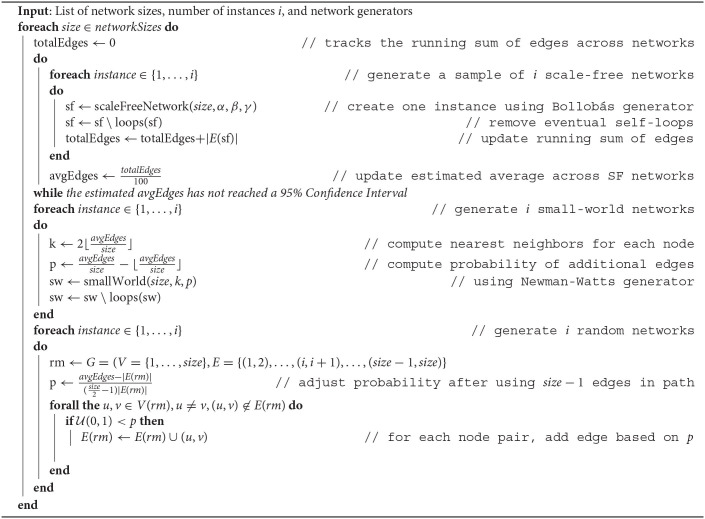
Generate comparable scale-free, small-world, and random networks for the desired sizes

## 4. Results

To support transparency and replicability of this work, we first explain where key results can be obtained by the readers (Section 4.1). We then confirm that comparable synthetic networks were generated across the three properties targeted in this paper (Section 4.2) and present our key results on time consumption as a function of network size (Section 4.3). **Table 4** summarizes the results.

### 4.1. Access to Results

Our main results are provided on the third party Open Science Framework at https://osf.io/4wy5c/. The timing results consist of the time and density for each centrality measure, each network type, each size, and each network instance. This produced 864, 000 measurements. The measurements were then visualized in Tableau through an extensive collection of plots; some of these visualizations are presented and discussed here. For full transparency, we also included each of the generated network instances on which we performed the measurements.

### 4.2. Validation of our Network Generation Procedure

To ensure that networks were similar in terms of edge density across types (random, scale-free, small-world), we measured the average and standard deviation of density at various network sizes. Table 3 confirms that our procedure for generating synthetic networks is valid, as it produced graphs with similar edge density for each network size. Although some variations are expected when using stochastic generators, the low standard deviation suggests that the edge density was similar across instances from each generator. We performed an (optional) search for the highest density, which was obtained for α = 0.07, β = 0.86, γ = 0.07. We note that an exhaustive search coupled with a large number of instances started to impose a noticeable computational load, thus this process required a high-performance computing cluster.

**Table 3:** Average edge densities of simulated network types for a sample of the sizes considered.

	**Network Type**			
**Network size**	**Scale-Free**	**Small-world**	**Random**	**St. Dev**.
100	0.03600	0.03593	0.03576	0.00010
200	0.01903	0.01900	0.01893	0.00005
400	0.01017	0.01031	0.01017	0.00006
800	0.00530	0.00558	0.00530	0.00013
1,600	0.00283	0.00316	0.00284	0.00015
3,200	0.00148	0.00170	0.00147	0.00011
6,400	0.00078	0.00093	0.00078	0.00007
12,800	0.00041	0.00050	0.00041	0.00004

### 4.3. Computational Performance

After collecting all centrality timing results from the High Performance Cluster, we computed the mean CPU time for each centrality metric on each metric type (across 100 instances). For each centrality metric and network type, the experimental data thus records the mean CPU time (y-axis) as a function of network size (x-axis). To identify the scaling relationship, we established which complexity function had the best fit among *log*(*n*), *n*, *nlog*(*n*), *n*^2^, *n*^2^*log*(*n*), and *n*^3^. Results across measures and network types are shown in Table 4.

**Table 4:** Experimental time complexities of 18 node centrality metrics on 3 network types.

**Metric**	**Network type**		
	**Scale-free**	**Small-world**	**Random**
Subgraph	*O*(*n*^2^)	*O*(*n*^2^)	*O*(*n*^2^*log*(*n*))
Geodesic K-Path	*O*(*n*^2^*log*(*n*))	*O*(*n*^2^)	*O*(*n*^2^*log*(*n*))
MNC	*O*(*nlog*(*n*))	*O*(*n*^2^)	*O*(*nlog*(*n*))
DMNC	*O*(*nlog*(*n*))	*O*(*n*^2^)	*O*(*nlog*(*n*))
Decay	*O*(*n*^2^*log*(*n*))	*O*(*n*^2^*log*(*n*))	*O*(*n*^2^*log*(*n*))
Topological	*O*(*n*^2^*log*(*n*))	*O*(*nlog*(*n*))	***O*(*n*)**
Lobby index	*O*(*nlog*(*n*))	*O*(*n*^2^)	*O*(*n*^2^)
Coreness	*O*(*nlog*(*n*))	*O*(*n*^2^)	*O*(*n*^3^)
Leverage	*O*(*nlog*(*n*))	*O*(*nlog*(*n*))	*O*(*nlog*(*n*))
Group	*O*(*nlog*(*n*))	*O*(*nlog*(*n*))	*O*(*nlog*(*n*))
Wiener Index	*O*(*n*^2^)	*O*(*n*^2^*log*(*n*))	*O*(*n*^2^*log*(*n*))
K-Path	*O*(*nlog*(*n*))	*O*(*nlog*(*n*))	*O*(*nlog*(*n*))
Diffusion degree	*O*(*nlog*(*n*))	*O*(*n*^2^)	*O*(*nlog*(*n*))
LeaderRank	*O*(*n*^2^)	*O*(*n*^2^)	*O*(*n*^3^)
Laplacian	*O*(*nlog*(*n*))	*O*(*n*^2^)	*O*(*nlog*(*n*))
Local bridging	*O*(*nlog*(*n*))	*O*(*nlog*(*n*))	*O*(*nlog*(*n*))
VoteRank	*O*(*n*^2^)	*O*(*n*^3^)	*O*(*n*^2^*log*(*n*))
HeatMap	*O*(*n*^2^*log*(*n*))	*O*(*n*^2^*log*(*n*))	*O*(*n*^2^*log*(*n*))

*Situations with good scaling behaviors are shown in green while those with a quickly rising cost are shown in red*.

**Figure 1:**
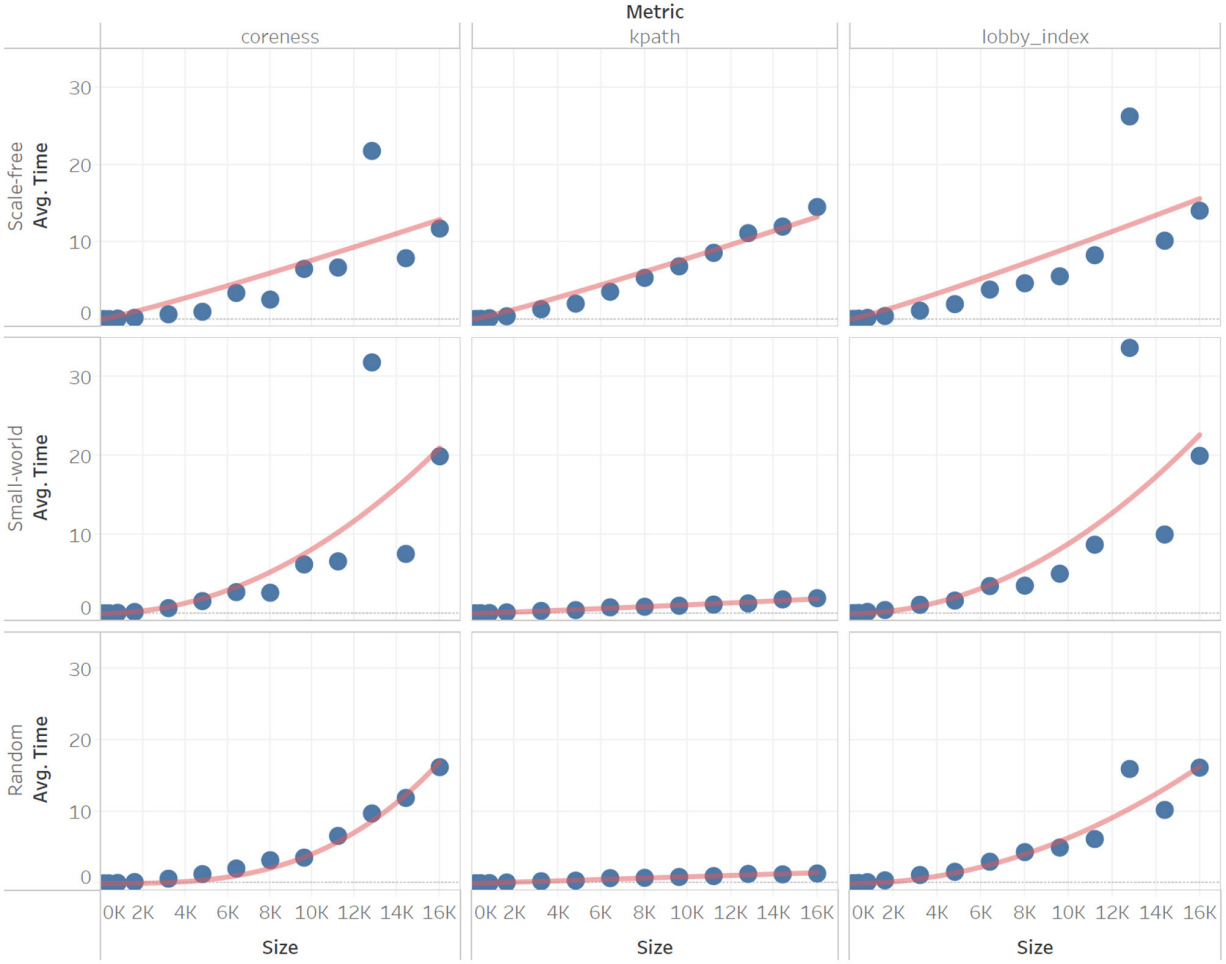
Sample of our experimental results on *Coreness, K-Path*, and *Lobby Index* for three network types (scale-free, small-world, random) of comparable network edges at varying network sizes (*n* = 100, 200, …, 16,000). Scaling is obtained by fitting on each column, for each network type.

**Figure 2:**
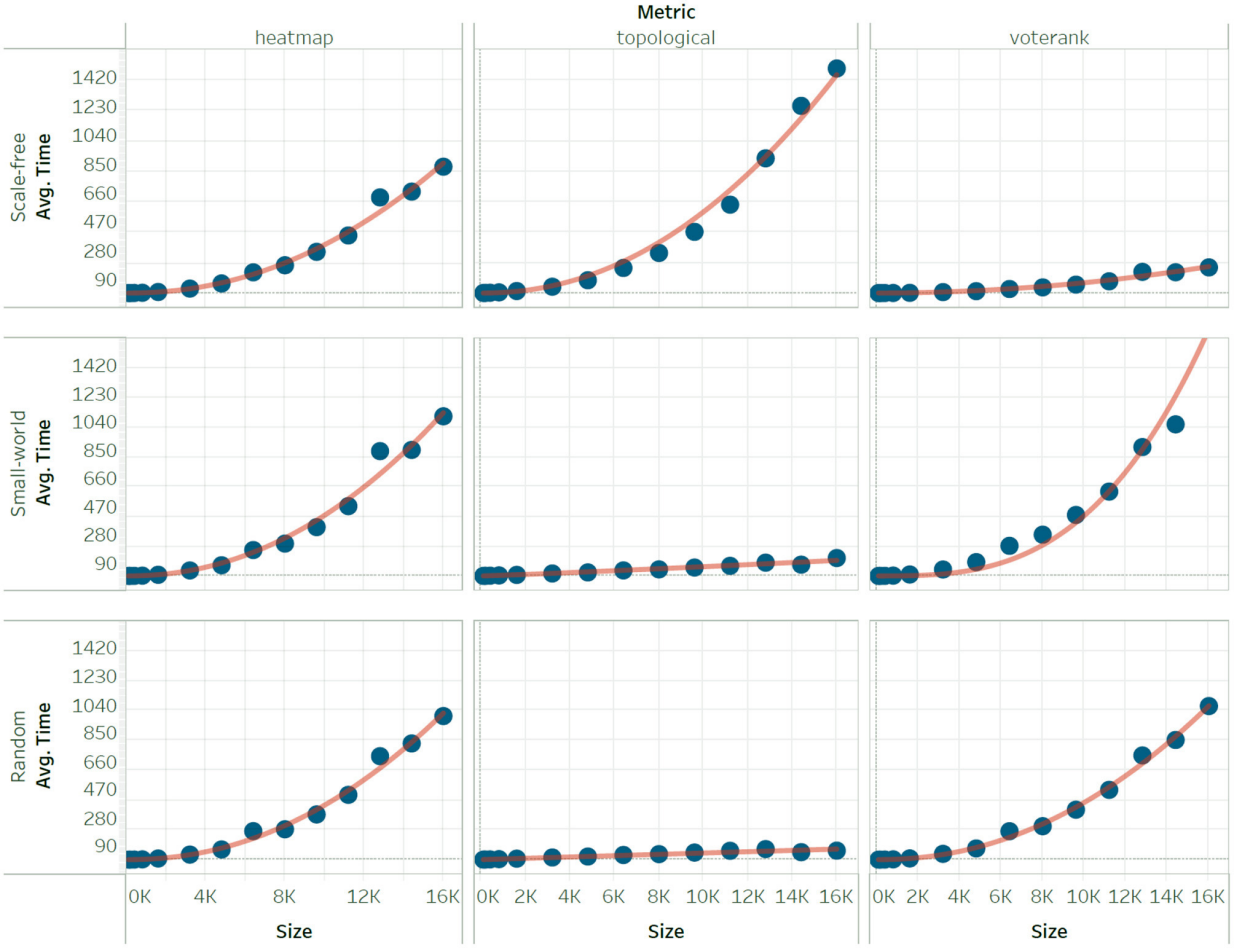
Sample of our experimental results on *Heatmap, Topological*, and *VoteRank* for three network types (scale-free, small-world, random) of comparable network edges at varying network sizes (*n* = 100, 200, …, 16,000). Scaling is obtained by fitting on each column, for each network type.

To further illustrate the approximation of time complexity, we also show how the function fits the empirical data for six measures. These illustration examples are divided based on scaling due to the wide difference on the scale of the y-axis. The group of measures that scale slowly (Figure 1) includes coreness, k-path, and the lobby index. The group of measures with a pronounced increased in CPU time (Figure 2) includes heatmap, topological, and voter rank.

## 5. Discussion

Beyond the classic measures such as betweenness or closeness, a plethora of measures continue to be proposed, fueled by the need to identify elements that bear particular importance in a given network type (e.g., brain networks) or as a means to complement existing measures. The time complexity to compute many of the classic centrality metrics is well-established, with known *O*(*nm*) solutions for betweenness and PageRank and *O*(*n* + *m*) for closeness and harmonic centrality. In contrast, the time complexity for many recent measures is less well understood, which may preclude their application to big network data, in which scalability is essential. Although estimating the worst case time complexity is feasible for several algorithms, their performance on real-world big network data may be drastically different from their theoretical worst case analysis. Consequently, our aim was to characterize the computational time necessary to perform 18 recent node centrality measures on large networks with commonly encountered properties such as scale-free or small-world.

Our results show that several centrality measures can cope with large network-sizes (Table 4–green), which makes them suitable for big network data as well as potential inclusion for composite measures or “centrality hybridization” (Singh et al., 2020). Under the current algorithm and implementation, several other measures (Table 4–red) would be limited to smaller networks, which makes them a prime target for follow-up studies using either distributed and/or approximation algorithms to deliver computational improvements. Such studies have a rich history in network science and continue to deliver improvements for well-known algorithms, as exemplified by ongoing works on parallel algorithms for degree or betweenness centrality (García and Carriegos, 2019), and approximation algorithms for Katz centrality (Lin et al., 2021). Our work thus provides the research community with a potential roadmap to improve emerging centrality measures by leveraging existing techniques. A possible starting point would be to *map these emerging measures to the most closely related classic centrality metrics*, and then assess whether established techniques for scalability can be reused with only minimal adjustments. Intuitively, we could expect that such a mapping may be facilitated by the wealth of empirical studies that employ several classic centrality metrics alongside a handful of newer metrics and show correlations either between centrality measures (Lavin et al., 2018) or with an application-dependent variable (Baniukiewicz et al., 2018). That is, researchers would be able to see that a newer metric is highly correlated with classic ones, and then attempt to reuse scalability techniques that worked on the classic measures. However, such correlation plots occasionally show that new metrics form isolated clusters, which bear no relation with classic measures (Zanghieri et al., 2021). Similar results were found using the less common approach of comparing measures *via* benchmarks: classic measures behaved similarly, whereas newer measures formed their own group (Bao and Zhang, 2021). The matter of re-using techniques for scalable implementations by relating newer measures to older ones is thus an open problem of its own.

We also briefly note that there is a strong research interest in GPU graphs analytics. The abundance of GPUs and their potential to speed up centrality measures was already noted over 10 years ago by Sharma et al. (2011). GPUs demonstrated their potential as hardware accelerators for classic metrics such as betweenness centrality (McLaughlin and Bader, 2018) or Eigenvector centrality (Sharma et al., 2011). More recently, this potential has been confirmed in the case of relatively newer measures such as the lobby index (Xiao et al., 2020).

We note that the cost of these centrality metrics is not solely a function of the number of nodes, but also of the structure of the network. This effect can be seen within a single metric, for example Coreness is in *O*(*n*^3^) on random networks but faster on small-world networks and even more on scale-free networks. One network type is not *systematically* worst than another for performances. For instance, topological centrality is more expensive than VoteRank on a scale-free network, but noticeably more efficient on small-world networks (*O*(*nlog*(*n*)) vs. *O*(*n*^3^). This shows that while network *size* has a predictable effect on centrality cost, and the only uncertainty is its magnitude, network *type* is far less predictable, and can even have opposite effects on a pair of metrics. In some cases, we found network type is so significant that it vastly exceeds the effect of network size on centrality performance. For example, the mean cost of K-Path Centralit in a Scale-Free Network of 3, 200 nodes is higher than the mean cost in a Small-World Network of 12, 800 nodes. Our results thus suggest that network type should be a common factor provided alongside time complexity for research involving centrality, as we have shown it is capable of having an equivalent effect on performance as network size.

Identifying whether a network exhibits certain common properties, such as Scale-Free or Small-World, is generally feasible for large networks (Barabasi, 2014), and even attainable in linear time for variations of these properties (Li et al., 2005; Humphries and Gurney, 2008; Zhou et al., 2020). In addition, analysts may already know whether their networks have certain properties, given the domain of application or familiarity with similar datasets. Our results thus enable researchers to identify metrics that have desired scaling behaviors given a target network size and type.

There are two main limitations to this research. First, our article is devoted to *sparse* complex networks. As shown in Table 2, all networks used in experimental studies for the centrality metrics considered here are sparse. However, this may have reflected a historical view of network science regarding scale-free networks, which were thought to all be sparse (Del Genio et al., 2011) and were usually generated using a preferential attachment mechanism. However, there is increased theoretical (Ma et al., 2020) and empirical evidence (Courtney and Bianconi, 2018) (e.g., from online social networks and brain networks) that scale-free networks *can be dense*. Several generators have thus been recently proposed to create dense scale-free networks (Courtney and Bianconi, 2018; Haruna and Gunji, 2019, 2020). It is thus possible that the next wave of centrality measures will be evaluated on a broader set of networks, including both sparse and dense cases. Consequently, their execution time may not be solely expressed as a function of the number of nodes, as done here for the case of sparse networks. Experiments on scalability could thus use the newer generators to produce scale-free graphs for a broad range of number of nodes *and* number of edges. Small-world generators and random generators can already produce such networks. Such updated experimental procedure will allow to report scalability as a function of the number of nodes and edges.

Second, we focused on three common archetypes of networks: small-world, scale-free, and random. In practice, there is a multiplicity of other network types. For example, networks may be polarized (Lofdahl et al., 2015), hierarchical (Giabbanelli, 2011), planar (Giabbanelli, 2010), or dynamic. This diversity is exemplified in generators of dynamic networks, which use methods as varied as stochastic blockmodels (Kim et al., 2018), graph neural networks (Skardinga et al., 2021), or event models (Fritz et al., 2020). A potential follow-up study could thus extend our approach by including network properties to assess whether they have pronounced effects on the cost of computing various measures. In turn, this may require the identification of suitable network generators and the development of a coherent procedure to create comparable networks across diverse generators.

## Data Availability Statement

The original contributions presented in the study are publicly available. This data can be found here: https://osf.io/4wy5c/.

## Author Contributions

The study was designed and supervised by PG. PG and AF jointly determined the methods and jointly wrote and approved the manuscript. AF wrote the code, produced, and analyzed the results.

## Funding

The authors wish to thank the Department of Computer Science and Software Engineering at Miami University for supporting AF with a graduate assistantship.

## Conflict of Interest

The authors declare that the research was conducted in the absence of any commercial or financial relationships that could be construed as a potential conflict of interest.

## Publisher's Note

All claims expressed in this article are solely those of the authors and do not necessarily represent those of their affiliated organizations, or those of the publisher, the editors and the reviewers. Any product that may be evaluated in this article, or claim that may be made by its manufacturer, is not guaranteed or endorsed by the publisher.

## References

[B1] Alahakoon T. Tripathi R. Kourtellis N. Simha R. Iamnitchi A. (2011). K-path centrality: a new centrality measure in social networks, in Proceedings of the 4th Workshop on Social Network Systems (Salzburg, Austria), 1–6.

[B2] Ali (2014). Big data 2014: The fourth ASE international conference big data, in Proceedings of the 2014 ASE BigData/SocialInformatics/PASSAT/BioMedCom 2014 Conference (Cambridge, MA: Harvard University). Available online at: https://www.iq.harvard.edu/files/iqss-harvard/files/conferenceprogram.pdf

[B3] Amblard F. Bouadjio-Boulic A. Gutiérrez C. S. Gaudou B. (2015). Which models are used in social simulation to generate social networks? a review of 17 years of publications in jasss, in 2015 Winter Simulation Conference (WSC) (Huntington Beach, CA: IEEE), 4021–4032.

[B4] Ashtiani M. Salehzadeh-Yazdi A. Razaghi-Moghadam Z. Hennig H. Wolkenhauer O. Mirzaie M.. (2018). A systematic survey of centrality measures for protein-protein interaction networks. BMC Syst. Biol. 12, 1–17. 10.1186/s12918-018-0598-2 30064421 PMC6069823

[B5] Baniukiewicz M. Dick Z. L. Giabbanelli P. J. (2018). Capturing the fast-food landscape in england using large-scale network analysis. EPJ Data Sci. 7, 39. 10.1140/epjds/s13688-018-0169-1 30956929 PMC6413857

[B6] Bao Q. Zhang Z. (2021). Discriminating power of centrality measures in complex networks. IEEE Trans. Cybern. 10.1109/TCYB.2021.3069839 33961577

[B7] Barabasi A.-L. (2014). Network Science: The Scale-Free Property. Available online at: https://barabasi.com/f/623.pdf

[B8] Barriere L. Comellas F. Dalfo C. Fiol M. A. (2016). Deterministic hierarchical networks. J. Phys. A 49, 225202. 10.1088/1751-8113/49/22/225202

[B9] Beauchamp M. A. (1965). An improved index of centrality. Behav. Sci. 10, 161–163. 10.1002/bs.3830100205 14284290

[B10] Besta M. Stanojevic D. Zivic T. Singh J. Hoerold M. Hoefler T. (2018). Log (graph) a near-optimal high-performance graph representation, in Proceedings of the 27th International Conference on Parallel Architectures and Compilation Techniques (Limassol, Cyprus), 1–13.

[B11] Bollobás B. Borgs C. Chayes J. T. Riordan O. (2003). Directed scale-free graphs, in SODA (Baltimore, MD, United States), Vol. 3, 132–139.

[B12] Borgatti S. P. Everett M. G. (2006). A graph-theoretic perspective on centrality. Soc. Netw. 28, 466–484. 10.1016/j.socnet.2005.11.00514531933

[B13] Borgatti S. P. Everett M. G. (2020). Three Perspectives on Centrality, Chapter 17. Oxford: Oxford University Press, 334–351.

[B14] Bringmann L. F. Elmer T. Epskamp S. Krause R. W. Schoch D. Wichers M.. (2019). What do centrality measures measure in psychological networks? J. Abnorm. Psychol. 128, 892. 10.1037/abn0000446 31318245

[B15] Campiteli M. G. Holanda A. J. Soares L. D. Soles P. R. Kinouchi O. (2013). Lobby index as a network centrality measure. Physica A 392, 5511–5515. 10.1016/j.physa.2013.06.065

[B16] Caporossi G. Paiva M. Vukičevic D. Segatto M. (2012). Centrality and betweenness: vertex and edge decomposition of the wiener index. MATCH Commun. Math. Comput. Chem. 68, 293–302.

[B17] Courtney O. T. Bianconi G. (2018). Dense power-law networks and simplicial complexes. Phys. Rev. E 97, 052303. 10.1103/PhysRevE.97.052303 29906951

[B18] Crofts J. J. Higham D. J. (2009). A weighted communicability measure applied to complex brain networks. J. R. Soc. Interface 6, 411–414. 10.1098/rsif.2008.0484 19141429 PMC2658663

[B19] Das K. Samanta S. Pal M. (2018). Study on centrality measures in social networks: a survey. Soc. Netw. Anal. Min. 8, 1–11. 10.1007/s13278-018-0493-2

[B20] Del Genio C. I. Gross T. Bassler K. E. (2011). All scale-free networks are sparse. Phys. Rev. Lett. 107, 178701. 10.1103/PhysRevLett.107.178701 22107590

[B21] Durón C. (2020). Heatmap centrality: a new measure to identify super-spreader nodes in scale-free networks. PLoS ONE 15, e0235690. 10.1371/journal.pone.0235690 32634158 PMC7340304

[B22] Estrada E. Rodriguez-Velazquez J. A. (2005). Subgraph centrality in complex networks. Phys. Rev. E 71, 056103. 10.1103/PhysRevE.71.056103 16089598

[B23] Everett M. Borgatti S. P. (2005). Ego network betweenness. Soc. Netw. 27, 31–38. 10.1016/j.socnet.2004.11.007

[B24] Freeman L. C. (1977). A set of measures of centrality based on betweenness. Sociometry 40, 35–41. 10.2307/3033543

[B25] Fritz C. Lebacher M. Kauermann G. (2020). Tempus volat, hora fugit: a survey of tie-oriented dynamic network models in discrete and continuous time. Stat. Neerl. 74, 275–299. 10.1111/stan.12198

[B26] Fronzetti Colladon A. Naldi M. (2020). Distinctiveness centrality in social networks. PLoS ONE 15, e0233276. 10.1371/journal.pone.0233276 32442196 PMC7244137

[B27] García J. F. Carriegos M. V. (2019). On parallel computation of centrality measures of graphs. J. Supercomput. 75, 1410–1428. 10.1007/s11227-018-2654-5

[B28] Giabbanelli P. J. (2010). Impact of complex network properties on routing in backbone networks, in 2010 IEEE Globecom Workshops (Miami, FL: IEEE), 389–393.

[B29] Giabbanelli P. J. (2011). The small-world property in networks growing by active edges. Adv. Complex Syst. 14, 853–869. 10.1142/S0219525911003207

[B30] Giabbanelli P. J. Jackson P. J. Finegood D. T. (2014). Modelling the joint effect of social determinants and peers on obesity among canadian adults, in Theories and Simulations of Complex Social Systems (Berlin; Heidelberg: Springer), 145–160.

[B31] Goyal P. Raja S. Huang D. Chhetri S. R. Canedo A. Mondal A.. (2020). Graph representation ensemble learning, in 2020 IEEE/ACM International Conference on Advances in Social Networks Analysis and Mining (ASONAM) (The Hague: IEEE), 24–31.

[B32] Gutman I. Zhou B. (2006). Laplacian energy of a graph. Linear Algebra Appl. 414, 29–37. 10.1016/j.laa.2005.09.008

[B33] Hage P. Harary F. (1995). Eccentricity and centrality in networks. Soc. Netw. 17, 57–63. 10.1016/0378-8733(94)00248-9

[B34] Haruna T. Gunji Y.-P. (2019). Ordinal preferential attachment: a self-organizing principle generating dense scale-free networks. Sci. Rep. 9, 1–8. 10.1038/s41598-019-40716-1 30858504 PMC6412141

[B35] Haruna T. Gunji Y.-P. (2020). Analysis and synthesis of a growing network model generating dense scale-free networks via category theory. Sci. Rep. 10, 1–8. 10.1038/s41598-020-79318-7 33339877 PMC7749186

[B36] Humphries M. D. Gurney K. (2008). Network “small-world-ness”: a quantitative method for determining canonical network equivalence. PLoS ONE 3, e0002051. 10.1371/journal.pone.0002051 18446219 PMC2323569

[B37] Jackson M. O. (2010). Social and Economic Networks. Princeton, NJ: Princeton University Press.

[B38] Jalili M. Salehzadeh-Yazdi A. Gupta S. Wolkenhauer O. Yaghmaie M. Resendis-Antonio O.. (2016). Evolution of centrality measurements for the detection of essential proteins in biological networks. Front. Physiol. 7, 375. 10.3389/fphys.2016.00375 27616995 PMC4999434

[B39] Jamour F. Skiadopoulos S. Kalnis P. (2017). Parallel algorithm for incremental betweenness centrality on large graphs. IEEE Trans. Parallel Distribut. Syst. 29, 659–672. 10.1109/TPDS.2017.276395127295638

[B40] Jones P. J. Ma R. McNally R. J. (2021). Bridge centrality: a network approach to understanding comorbidity. Multivariate Behav. Res. 56, 353–367. 10.1080/00273171.2019.1614898 31179765

[B41] Joyce K. E. Laurienti P. J. Burdette J. H. Hayasaka S. (2010). A new measure of centrality for brain networks. PLoS ONE 5, e12200. 10.1371/journal.pone.0012200 20808943 PMC2922375

[B42] Kanovsky I. (2010). Small world models for social network algorithms testing. Procedia Comput. Sci. 1, 2341–2344. 10.1016/j.procs.2010.04.26325851082

[B43] Katz L. (1953). A new status index derived from sociometric analysis. Psychometrika 18, 39–43. 10.1007/BF02289026

[B44] Keng Y. Y. Kwa K. H. McClain C. (2021). Convex combinations of centrality measures. J. Math. Sociol. 45, 195–222. 10.1080/0022250X.2020.1765776

[B45] Kim B. Lee K. H. Xue L. Niu X. (2018). A review of dynamic network models with latent variables. Stat. Surv. 12, 105. 10.1214/18-SS121 31428219 PMC6699782

[B46] Kitsak M. Gallos L. K. Havlin S. Liljeros F. Muchnik L. Stanley H. E.. (2010). Identification of influential spreaders in complex networks. Nat. Phys. 6, 888–893. 10.1038/nphys174630093716

[B47] Koschützki D. Lehmann K. A. Peeters L. Richter S. Tenfelde-Podehl D. Zlotowski O. (2005). Centrality indices, in Network Analysis (Berlin; Heidelberg: Springer), 16–61.

[B48] Kundu S. Murthy C. Pal S. K. (2011). A new centrality measure for influence maximization in social networks, in International Conference on Pattern Recognition and Machine Intelligence (Berlin; Heidelberg: Springer), 242–247.

[B49] Lavin E. A. Giabbanelli P. J. Stefanik A. T. Gray S. A. Arlinghaus R. (2018). Should we simulate mental models to assess whether they agree? in Proceedings of the Annual Simulation Symposium (Baltimore, MD, United States), 1–12.

[B50] Li L. Alderson D. Doyle J. C. Willinger W. (2005). Towards a theory of scale-free graphs: definition, properties, and implications. Internet Math. 2, 431–523. 10.1080/15427951.2005.10129111

[B51] Lin C.-Y. Chin C.-H. Wu H.-H. Chen S.-H. Ho C.-W. Ko M.-T. (2008). Hubba: hub objects analyzer–a framework of interactome hubs identification for network biology. Nucleic Acids Res. 36(suppl_2):W438–W443. 10.1093/nar/gkn257 18503085 PMC2447731

[B52] Lin M. Li W. Song L. J. Nguyen C.-T. Wang X. Lu S. (2021). Sake: Estimating katz centrality based on sampling for large-scale social networks. ACM Trans. Knowl. Discov. Data 15, 1–21. 10.1145/3441646

[B53] Lofdahl C. Stickgold E. Skarin B. Stewart I. (2015). Extending generative models of large scale networks. Procedia Manufact. 3, 3868–3875. 10.1016/j.promfg.2015.07.89633481712

[B54] Lü L. Zhang Y.-C. Yeung C. H. Zhou T. (2011). Leaders in social networks, the delicious case. PLoS ONE 6, e21202. 10.1371/journal.pone.0021202 21738620 PMC3124485

[B55] Ma F. Wang X. Wang P. (2020). Scale-free networks with invariable diameter and density feature: counterexamples. Phys. Rev. E 101, 022315. 10.1103/PhysRevE.101.022315 32168588

[B56] Macker J. P. (2016). An improved local bridging centrality model for distributed network analytics, in MILCOM 2016-2016 IEEE Military Communications Conference, (Baltimore, MD: IEEE), 600–605.

[B57] Matta J. Ercal G. Sinha K. (2019). Comparing the speed and accuracy of approaches to betweenness centrality approximation. Comput. Soc. Netw. 6, 1–30. 10.1186/s40649-019-0062-5

[B58] McLaughlin A. Bader D. A. (2018). Accelerating gpu betweenness centrality. Commun. ACM. 61, 85–92. 10.1145/3230485

[B59] Mei A. Stefa J. (2009). Swim: a simple model to generate small mobile worlds, in IEEE INFOCOM 2009 (Rio de Janeiro: IEEE), 2106–2113.

[B60] Mnasri W. Azaouzi M. Romdhane L. B. (2021). Parallel social behavior-based algorithm for identification of influential users in social network. Appl. Intell. 51, 7365–7383. 10.1007/s10489-021-02203-x 34764589 PMC7938287

[B61] Narayanam R. Narahari Y. (2010). A shapley value-based approach to discover influential nodes in social networks. IEEE Trans. Autom. Sci. Eng. 8, 130–147. 10.1109/TASE.2010.205204227295638

[B62] Newman M. E. Watts D. J. (1999). Renormalization group analysis of the small-world network model. Phys. Lett. A 263, 341–346. 10.1016/S0375-9601(99)00757-4

[B63] Oldham S. Fulcher B. Parkes L. Arnatkeviciute A. Suo C. Fornito A. (2019). Consistency and differences between centrality measures across distinct classes of networks. PLoS ONE 14, e0220061. 10.1371/journal.pone.0220061 31348798 PMC6660088

[B64] Pasta Q. Zaidi F. (2018). Model to generate benchmark graphs based on evolution dynamics, in 2018 IEEE/ACM International Conference on Advances in Social Networks Analysis and Mining (ASONAM) (Barcelona: IEEE), 1223–1231.

[B65] Qin Z. Yu T. Yang Y. Khalil I. Xiao X. Ren K. (2017). Generating synthetic decentralized social graphs with local differential privacy, in Proceedings of the 2017 ACM SIGSAC Conference on Computer and Communications Security (Dallas, Texas, USA), 425–438.

[B66] Ravasz E. Barabási A.-L. (2003). Hierarchical organization in complex networks. Phys. Rev. E 67, 026112. 10.1103/PhysRevE.67.026112 12636753

[B67] Rossetti G. (2017). Rdyn: graph benchmark handling community dynamics. J. Complex Netw. 5, 893–912. 10.1093/comnet/cnx016

[B68] Sahraeian S. M. E. Yoon B.-J. (2012). A network synthesis model for generating protein interaction network families. PLoS ONE 7, e41474. 10.1371/journal.pone.0041474 22912671 PMC3418285

[B69] Salavaty A. Ramialison M. Currie P. D. (2020). Integrated value of influence: an integrative method for the identification of the most influential nodes within networks. Patterns 1, 100052. 10.1016/j.patter.2020.100052 33205118 PMC7660386

[B70] Saxena R. Jadeja M. (2022). Network centrality measures: Role and importance in social networks, in Principles of Social Networking (Berlin; Heidelberg: Springer), 29–54.

[B71] Sharma P. Khurana U. Shneiderman B. Scharrenbroich M. Locke J. (2011). Speeding up network layout and centrality measures for social computing goals, in International Conference on Social Computing, Behavioral-Cultural Modeling, and Prediction (Berlin; Heidelberg: Springer), 244–251.

[B72] Singh A. Singh R. R. Iyengar S. (2020). Node-weighted centrality: a new way of centrality hybridization. Comput. Soc. Netw. 7, 1–33. 10.1186/s40649-020-00081-w

[B73] Singh R. R. (2022). Centrality measures: a tool to identify key actors in social networks, in Principles of Social Networking (Berlin; Heidelberg: Springer), 1–27.

[B74] Skardinga J. Gabrys B. Musial K. (2021). Foundations and modelling of dynamic networks using dynamic graph neural networks: a survey. IEEE Access. 9, 79143–79168. 10.1109/ACCESS.2021.308293227295638

[B75] Tew K. L. Li X.-L. Tan S.-H. (2007). Functional centrality: detecting lethality of proteins in protein interaction networks. Genome Inform. 19, 166–177. 10.1142/9781860949852_0015 18546514

[B76] Ugander J. Karrer B. Backstrom L. Marlow C. (2011). The anatomy of the facebook social graph. CoRR abs/1111.4503.

[B77] van der Grinten A. Angriman E. Meyerhenke H. (2020). Scaling up network centrality computations-a brief overview. it-Information Technol. 62, 189–204. 10.1515/itit-2019-0032

[B78] van der Grinten A. Angriman E. Predari M. Meyerhenke H. (2021). New approximation algorithms for forest closeness centrality-for individual vertices and vertex groups, in Proceedings of the 2021 SIAM International Conference on Data Mining (SDM) (SIAM) (Online), 136–144.

[B79] Wang S. Mei G. Cuomo S. (2019). A simple and generic paradigm for creating complex networks using the strategy of vertex selecting-and-pairing. Future Gen. Comput. Syst. 100, 994–1004. 10.1016/j.future.2019.05.071

[B80] Xiao L. Wang S. Mei G. (2020). Efficient parallel algorithm for detecting influential nodes in large biological networks on the graphics processing unit. Future Gen. Comput. Syst. 106, 1–13. 10.1016/j.future.2019.12.038

[B81] Yang X.-H. Chen G. Chen S.-Y. (2013). The impact of connection density on scale-free distribution in random networks. Physica A 392, 2547–2554. 10.1016/j.physa.2013.01.038

[B82] Zanghieri M. Menichetti G. Retico A. Calderoni S. Castellani G. Remondini D. (2021). Node centrality measures identify relevant structural mri features of subjects with autism. Brain Sci. 11, 498. 10.3390/brainsci11040498 33919984 PMC8071038

[B83] Zarghami S. A. Gunawan I. (2019). A domain-specific measure of centrality for water distribution networks. Eng. Construct. Arch. Manag. 27, 341–355. 10.1108/ECAM-03-2019-0176

[B84] Zhang J.-X. Chen D.-B. Dong Q. Zhao Z.-D. (2016). Identifying a set of influential spreaders in complex networks. Sci. Rep. 6, 27823. 10.1038/srep27823 27296252 PMC4906276

[B85] Zhou B. Meng X. Stanley H. E. (2020). Power-law distribution of degree-degree distance: a better representation of the scale-free property of complex networks. Proc. Natl. Acad. Sci. U.S.A. 117, 14812–14818. 10.1073/pnas.1918901117 32541015 PMC7334507

[B86] Zhuge H. Zhang J. (2009). Topological centrality and its applications. arXiv preprint arXiv:0902.1911.

