# Supplementary material for: An Experimental Study on the Scalability of Recent Node Centrality Metrics in Sparse Complex Networks
Source: Front Big Data. 2022 Feb 16;5:797584. doi: 10.3389/fdata.2022.797584 (PMC8889076; doi:10.3389/fdata.2022.797584)
Supplement: Supplementary file 1 [file Data_Sheet_1.PDF]

frontiers

Supplementary Material

1 EQUATIONS FOR RECENT NODE CENTRALITY MEASURES

Subgraph Centrality (2005) uses the set of eigenvalues of the adjacency matrix (the graph spectrum) to count the number of closed walks of length k from a given node.

$$C_{SG}(v) = \sum_{k=0}^{\infty} \frac{[A^k]_{vv}}{k!} \cdot 1 = [e^A]_{vv} \cdot 1 \tag{S1}$$

where $[A^k]_{vv}$ is the number of closed walks of length k starting and ending at node v, e^A is the exponential of the adjacency matrix, and 1 is the vector of all ones (Estrada and Rodriguez-Velazquez, 2005).

Geodesic K-Path Centrality (2006) for a given node v is equal to the number of nodes u_i such that the shortest path from v to u_i is at most k. By default, we use $k = 3$ (Borgatti and Everett, 2006).

$$C_{GKP}(v) = |\forall v_i \in V : d(v, v_i) \leq k| \tag{S2}$$

Maximum Neighborhood Component Centrality (2008) is equal to the size of the largest connected component within the direct neighbors of a given node, not including the node itself.

$$C_{MNC}(v) = |MC(N(v))| \tag{S3}$$

where $MC(N(v))$ is the largest connected component within the neighbors of v (Lin et al., 2008).

Density of Maximum Neighborhood Component Centrality (2008) is defined as the ratio of edges to nodes within the largest connected component between a node's neighbors.

$$C_{DMNC}(v) = \frac{|E(MNC(v)|}{|V(MNC(v))|^{\epsilon}} \tag{S4}$$

where ϵ is a density parameter such that $1 \leq \epsilon \leq 2$ (Lin et al., 2008).

Decay Centrality (2008) is based on the proximity between a given node and every other node, weighted by a decay rate.

$$C_{Decay}(v) = \sum_{u \in V(G)} \eta^{d(v,u)} \tag{S5}$$

where $\eta \in [0, 1]$ is the decay rate (Jackson, 2010).

Topological Coefficient Centrality (2009) is equal to the average number of neighbors of a given node that are also neighbors to a different node.

$$C_T(v) = \frac{avg(J(v,u))}{|N(v)|} \forall u \in V : |N(u) \cap N(v)| > 0 \tag{S6}$$

where u is a node that shares at least one neighbor with v, and $J(v, u)$ is the number of neighbors shared by v and u (Zhuge and Zhang, 2009).

Lobby Index Centrality (2009) for a given node is equal to the largest integer k such that the node has at least k neighbors with a degree of at least k.

$$C_{LI}(v) = max(k) : k \geq |N(v)| \wedge k \geq \Delta(N(v)) \tag{S7}$$

where $\Delta(N(v))$ is the maximum degree of the neighbors of v (Campiteli et al., 2013).

Coreness Centrality (2010) is equal to the sum of k-shell indexes of a given node's neighbors, where the k-core of a graph is the maximal connected subgraph such that all nodes have degree at least k, and the k-shell is the set of nodes in the k-core that are not in the $k+1$-core.

$$C_{NC}(v) = \sum_{w \in N(v)} ks(w) \tag{S8}$$

where $ks(w)$ is the k-shell index of w (Kitsak et al., 2010).

Leverage Centrality (2010) is based on the degree of a node relative to its neighbors (Joyce et al., 2010).

$$C_{Lev}(v) = \frac{1}{deg(v)} \sum_{i \in N(v)} \frac{deg(v) - deg(i)}{deg(v) + deg(i)} \tag{S9}$$

Group Centrality (2010) is computed using the Shapley Value of a node, originating from the field of Game Theory. This value is used to estimate the importance of a player in a cooperative game, in order to determine their reward, but it can also be applied to graphs. The Shapley Value is a measurement of the marginal increase in group influence when a given node is added.

$$C_G(v) = \frac{1}{|V(G)|!} \sum_{\pi \in \Omega} f(C_\pi(v) \cup \{v\}) - f(C_\pi(v)) \tag{S10}$$

where Ω is the set of all possible permutations of nodes, $f(S)$ is a parameter that computes the influence of a subset S, and $C_\pi(v)$ is the set of all nodes appearing before v in the permutation π (Narayanam and Narahari, 2010).

Wiener Index Centrality (2011) is equal to the average distance from a given node to all other nodes in a graph. It is equivalent to the reciprocal of the Closeness Centrality (Caporossi et al., 2012).

$$C_{WI}(v) = \sum_{u \in G} d(v, u) \tag{S11}$$

K-Path Centrality (2011) is based on the number of random paths of length k originating from all possible source nodes that include a given node.

$$C_{KP}(v) = \sum_{s \in V} \frac{\sigma_s^k(v)}{\sigma_s^k} \tag{S12}$$

where s are all possible source nodes, $\sigma_s^k(v)$ is the number of k-paths originating from s and passing through v, and σ_s^k is the total number of k-paths originating from s (Alahakoon et al., 2011).

Diffusion Degree Centrality (2011) is based on the degree contribution of both a given node and its neighbors.

$$C_{DD}(v) = \lambda_v * deg(v) + \sum_{i \in N(v)} \lambda_i * deg(i) \tag{S13}$$

where λ_i is the propagation probability of node i, which is supplied as a parameter. The default value of λ is 1 for all nodes (Kundu et al., 2011).

LeaderRank Centrality (2011) is based on a random walk starting at a ground node, which is artificially connected to every node in the original network. The score of every node is initialized to 1, and incremented when the random walk visits that node. The ratio between node scores converges over time and is used to produce a centrality ranking. This metric is designed for directed graphs, but can be used on undirected graphs as well.

$$C_{Lead}(v_{t+1}) = \sum_{j=1}^{|V(G)|+1} \frac{a_{jv}}{deg^+(j)} C_{Lead}(j_t) \tag{S14}$$

where $deg^+(j)$ is the out degree of node j (Lü et al., 2011).

Laplacian Centrality (2011) is equal to the drop in Laplacian energy (Gutman and Zhou, 2006) when a given node is removed, or the sum of squares of the eigenvalues in the Laplacian matrix. It can be computed efficiently using just the degrees of a node and its neighbors (Qi et al., 2012).

$$C_{Lap}(v) = deg^2(v) + deg(v) + 2 \sum_{v_i \in N(v)} deg(v_i) \tag{S15}$$

Local Bridging Centrality (2016) defines a bridge in a network as a node that is located between (and connects) modules. Unlike the existing Bridging Centrality (Hwang et al., 2008), this measurement computes a score based solely on local metrics, namely the local Betweenness Centrality, degree, and a bridging coefficient.

$$C_{LB}(v) = C_{lbet}(v) * \beta_c(v) = \sum_{s \neq v \neq r} \frac{\sigma_{sr}(v)}{\sigma_{sr}} * \frac{\frac{1}{deg(v)}}{\sum_{i \in N(v)} \frac{1}{deg(i)}} \tag{S16}$$

where s and r are neighbors of v and $\beta_c(v)$ is the bridging coefficient of v (Macker, 2016).

VoteRank Centrality (2016) is based on rounds of simultaneous voting in which nodes elect their neighbors. Then, the converged scores are used for ranking and to identify decentralized nodes with the best spreading ability.

$$C_{VR}(v) = i \iff v = R_i : \forall u \in V, (u_s, u_a)_t = \begin{cases} (0,1), & t = 0, \\ (0,0), u \to R, & u = u_{max}, \\ (\sum_{w \in N(u), w \notin R} w_a, u_a - \frac{1}{k}) & u_{max} \in N(u), \\ (\sum_{w \in N(u), w \notin R} w_a, u_a) & otherwise. \end{cases} \tag{S17}$$

where $(u_s, u_a)_t$ are the voting score and ability of u at time t, $u \to R$ refers to adding u to the sequence R, u_{max} is the node with the highest increase of u_s in the current round, and k is average network degree (Zhang et al., 2016).

Heatmap Centrality (2020) is computed using the farness (inverse of closeness) of all nodes from a given node and the average farness from each of that node's neighbors.

$$C_{HM}(v_i) = \sum_{j=1}^{N} f(v_i, v_j) - \frac{\sum_{j=1}^{N} a_{ij} \cdot \sum_{k=1}^{N} f(v_j, v_k)}{\sum_{j=1}^{N} a_{ij}} \tag{S18}$$

where $f(v_i, v_j)$ is the farness between v_i and v_j, and a_{ij} is the weight of the edge from v_i to v_j (Durón, 2020).

REFERENCES

Alahakoon, T., Tripathi, R., Kourtellis, N., Simha, R., and Iamnitchi, A. (2011). K-path centrality: A new centrality measure in social networks. In *Proceedings of the 4th workshop on social network systems*. 1–6

Banerjee, A., Chandrasekhar, A. G., Duflo, E., and Jackson, M. O. (2013). The diffusion of microfinance. *Science* 341

Benzi, M. and Klymko, C. (2013). Total communicability as a centrality measure. *Journal of Complex Networks* 1, 124–149

Borgatti, S. P. and Everett, M. G. (2006). A graph-theoretic perspective on centrality. *Social networks* 28, 466–484

Campiteli, M. G., Holanda, A. J., Soares, L. D., Soles, P. R., and Kinouchi, O. (2013). Lobby index as a network centrality measure. *Physica A: Statistical Mechanics and its Applications* 392, 5511–5515

Caporossi, G., Paiva, M., Vukičevic, D., and Segatto, M. (2012). Centrality and betweenness: vertex and edge decomposition of the wiener index. *MATCH-Communications in Mathematical and Computer Chemistry* 68, 293

Chen, D., Lü, L., Shang, M.-S., Zhang, Y.-C., and Zhou, T. (2012). Identifying influential nodes in complex networks. *Physica a: Statistical mechanics and its applications* 391, 1777–1787

Chen, D.-B., Gao, H., Lü, L., and Zhou, T. (2013). Identifying influential nodes in large-scale directed networks: the role of clustering. *PloS one* 8, e77455

Durón, C. (2020). Heatmap centrality: A new measure to identify super-spreader nodes in scale-free networks. *Plos one* 15, e0235690

Estrada, E. and Rodriguez-Velazquez, J. A. (2005). Subgraph centrality in complex networks. *Physical Review E* 71, 056103

Gutman, I. and Zhou, B. (2006). Laplacian energy of a graph. *Linear Algebra and its applications* 414, 29–37

Hwang, W., Kim, T., Ramanathan, M., and Zhang, A. (2008). Bridging centrality: graph mining from element level to group level. In *Proceedings of the 14th ACM SIGKDD international conference on Knowledge discovery and data mining*. 336–344

Jackson, M. O. (2010). *Social and economic networks* (Princeton university press)

Joyce, K. E., Laurienti, P. J., Burdette, J. H., and Hayasaka, S. (2010). A new measure of centrality for brain networks. *PloS one* 5, e12200

Kermarrec, A.-M., Le Merrer, E., Sericola, B., and Trédan, G. (2011). Second order centrality: Distributed assessment of nodes criticity in complex networks. *Computer Communications* 34, 619–628

Kitsak, M., Gallos, L. K., Havlin, S., Liljeros, F., Muchnik, L., Stanley, H. E., et al. (2010). Identification of influential spreaders in complex networks. *Nature physics* 6, 888–893

Kundu, S., Murthy, C., and Pal, S. K. (2011). A new centrality measure for influence maximization in social networks. In *International Conference on Pattern Recognition and Machine Intelligence* (Springer), 242–247

Lin, C.-Y., Chin, C.-H., Wu, H.-H., Chen, S.-H., Ho, C.-W., and Ko, M.-T. (2008). Hubba: hub objects analyzer—a framework of interactome hubs identification for network biology. *Nucleic acids research* 36, W438–W443

Lü, L., Zhang, Y.-C., Yeung, C. H., and Zhou, T. (2011). Leaders in social networks, the delicious case. *PloS one* 6, e21202

Macker, J. P. (2016). An improved local bridging centrality model for distributed network analytics. In *MILCOM 2016-2016 IEEE Military Communications Conference* (IEEE), 600–605

Narayanam, R. and Narahari, Y. (2010). A shapley value-based approach to discover influential nodes in social networks. *IEEE Transactions on Automation Science and Engineering* 8, 130–147

Qi, X., Fuller, E., Wu, Q., Wu, Y., and Zhang, C.-Q. (2012). Laplacian centrality: A new centrality measure for weighted networks. *Information Sciences* 194, 240–253

White, S. and Smyth, P. (2003). Algorithms for estimating relative importance in networks. In *Proceedings of the ninth ACM SIGKDD international conference on Knowledge discovery and data mining*. 266–275

Zhang, J.-X., Chen, D.-B., Dong, Q., and Zhao, Z.-D. (2016). Identifying a set of influential spreaders in complex networks. *Scientific reports* 6, 27823

Zhuge, H. and Zhang, J. (2009). Topological centrality and its applications. *arXiv preprint arXiv:0902.1911*
